# High Throughput Screening Platform for a FAD-Dependent L-Sorbose Dehydrogenase

**DOI:** 10.3389/fbioe.2020.00194

**Published:** 2020-03-17

**Authors:** Xiaoyu Shan, Li Liu, Weizhu Zeng, Jian Chen, Jingwen Zhou

**Affiliations:** ^1^School of Biotechnology and Key Laboratory of Industrial Biotechnology, Ministry of Education, Jiangnan University, Wuxi, China; ^2^National Engineering Laboratory for Cereal Fermentation Technology, Jiangnan University, Wuxi, China; ^3^The Key Laboratory of Carbohydrate Chemistry and Biotechnology, Ministry of Education, Jiangnan University, Wuxi, China; ^4^Jiangsu Provisional Research Center for Bioactive Product Processing Technology, Jiangnan University, Wuxi, China

**Keywords:** 2-Keto-L-gulonic acid, promoter, sorbose dehydrogenase, sorbosone dehydrogenase, aldosterone reductase, L-sorbose-specific-PTS, error-prone PCR

## Abstract

2-Keto-L-gulonic acid (2-KLG) is the direct precursor for the production of L-ascorbic acid (L-Asc) on industrial scale. Currently, the production of L-Asc in the industry is a two-step fermentation process. Owing to many unstable factors in the fermentation process, the conversion rate of L-sorbose to 2-KLG has remained at about 90% for many years. In order to further improve the production efficiency of 2-KLG, a FAD-dependent sorbose dehydrogenase (SDH) has been obtained in our previous research. The SDH can directly convert L-sorbose to 2-KLG at a very high efficiency. However, the enzyme activity of the SDH is relatively low. In order to further improve the enzyme activity of the SDH, a high throughput screening platform the dehydrogenase is essential. By optimizing the promoter, host and sorbosone dehydrogenase (SNDH), knockout of the aldosterone reductases and PTS related genes, a reliable platform for high-throughput screening of more efficient FAD-dependent SDH has been established. By using the high-throughput screening platform, the titer of the 2-KLG has been improved by 14.1%. The method established here could be useful for further enhancing the FAD-dependent SDH, which is important to achieve the efficient one-strain-single-step fermentation production of 2-KLG.

## Introduction

Vitamin C, also known as L-ascorbic acid (L-Asc), is an essential nutrient for human beings and occupies the largest global market among vitamins. It can affect cells by participating in the elimination of reactive oxygen species (Wenzel et al., 2004), promoting collagen production (May and Qu, 2005), and helping immune defense (Carr and Maggini, 2017). At present, the main L-Asc production method is the classical two-step fermentation process, which uses *Gluconobacter oxydans* to convert D-sorbitol to L-sorbose, and uses *Ketogulonigenium vulgare* and *Bacillus megaterium* to further convert L-sorbose to 2-keto-L-gulonic acid (2-KLG). The process has two fermentation steps and requires two times of sterilization, resulting in high energy consumption and long production period (Liu et al., 2011a,b). Besides, compared with commonly used single-fermentation fermentation, both regulation and breeding of mixed fermentation are more difficult (Kim et al., 2019). To achieve the one-step-single-strain production of 2-KLG is a long pursued goal for the vitamin C industry.

In order to achieve the one-step-single-strain production of 2-KLG, previous attempts could be divided into several groups: (1) Overexpression of sorbose dehydrogenase (SDH) and sorbosone dehydrogenase (SNDH) from *K. vulgare* in *G. oxydans* or other bacteria (Gao et al., 2014). Since the SDH from *K. vulgare* could also interact with D-sorbitol to form other byproducts, the yield of 2-KLG on D-sorbitol by this method remains to be lower than 65%. (2) Overexpression of 2,5-diketo-D-gluconate reductase from *Corynebacterium glutamicum* in *Erwinia sp*. that can product 2,5-diketo-D-gluconate from D-glucose (Anderson et al., 1985). For potential low enzyme activity or cofactor balance issues, 2-KLG titer could not be significantly improved by using the method. (3) Overexpression of SDH and SNDH from *G. oxydans* T100 in *G. oxydans* strains that can produce 2-KLG from D-sorbitol (Saito et al., 1997). When expressing the SDH gene from *G. oxydans* T100 in *E. coli*, the SDH cannot catalyze L- sorbose to 2-KLG, suggesting that it may be a major limiting step to increase the enzyme activity of this SDH. According to these previous reports, the main problems in achieving one-step-single-strain fermentation of 2-KLG include cofactor regeneration (Wang et al., 2016; Kim et al., 2019), identification of key enzymes, competition of intermediate metabolic byproducts (Richter et al., 2009).

In our recent report, we obtained a *G. oxydans* strain by high-throughput screening aided with 2-KLG dehydrogenase (Chen et al., 2019). A FAD-dependent SDH that can convert L-sorbose to 2-KLG with almost 100% conversion efficiency has been identified from the strain. However, the enzyme activity of the SDH is very low and can only produce several grams of 2-KLG by different optimization method. Based the previous reports about other SDHs that can convert L-sorbose to L-sorbosone or 2-KLG (Zhou et al., 2012; Gao et al., 2017), both substrate/product specificity and enzyme activity should be improved by high-throughput screening. Since the slow growth rate of *G. oxydans* and low transformation efficiency (Yao et al., 2017; Jin et al., 2019), *G. oxydans* itself is not suitable for high-throughput screening of efficient SDH. Therefore, it is essential to construct a high-throughput screening platform strain with fast growth and high transformation efficiency. Since the SDH could be functional expressed in *E. coli* to catalyze L-sorbose to 2-KLG, *E. coli* could be a good choice (Chen et al., 2019). Though D-sorbitol, L-sorbose, L-sorbosone and 2-KLG are not commonly acquired in *E. coli*, the aldosterone reductases and PTS-related proteins could affect the regular function of SDH and thus interrupt the high-throughput screening process.

This study was focused on establishment of a high-throughput screening platform of a FAD-dependent SDH from *G. oxydans* WSH-003 based on *E. coli*, and then improve the enzyme activity of the SDH. By optimizing the promoter of the SDH, it was found that introduction of SDH in *E. coli* not only produce 2-KLG, there are also some byproducts could be found. In order to achieve more reliable screening process, the aldosterone reductases and PTS related genes are knockout consequently, combined with the co-expression of a SNDH. By using the high-throughput screening system, the titer of the 2-KLG has been improved by 14.1%. The method established here could be useful for further enhancing the FAD-dependent SDH, which is important to achieve the efficient one-strain-single-step fermentation production of 2-KLG.

## Materials and Methods

### Genes, Plasmids, and Strains

*E. coli* JM109 was used for plasmid construction and preservation. *E. coli* BL21 (DE3) was used for gene expression research. The plasmids pCas and pTarget required for gene knockout were provided by Shanghai Institute of Plant Physiology and Ecology (Jiang et al., 2015). *G. oxydans* WSH-004 was screened in our previous research (Chen et al., 2019).

### Plasmid Construction and Gene Expression

Five constitutive promoters with different strength (P_infC−rplT_, P_lpp_, P_dnaKJ_, P_cspA_, P_csrA_) have been selected to express SDH (Zhou et al., 2017). These promoters were obtained by PCR amplification using the *E. coli* K12 genome as the template. SDH gene was PCR-amplified from the genomic DNA of *G. oxydans* WSH-004. The target gene SDH and these promoters were ligated into the vector pMD19-T-Simple by a one-step cloning kit (Takara, Dalian, China). All the primers used for expression of SDH are listed Table 1. These plasmids have also been transformed into *E. coli* K12substr. W3110, *E. coli* K12substr. MG1655, *E. coli* JM109 for selecting optimum *E. coli* host.

**Table 1:** Primers used for promoter optimization.

**Primer**	**Sequence 5^**′**^-3^**′**^**
infC-rplT-F	gcgcggatcttccagagattctgaatacgttaacgaattgacgc
infC-rplT-R	agttcttctcccttacccataccttattcctccaattgtttaagac
lpp-F	gcgcggatcttccagagatttgaatccgatggaagcatcctg
lpp-R	agttcttctcccttacccattattaataccctctagattgagttaatctc
cspA-F	cgcggatcttccagagattattgctgtttacggtcctgatg
cspA-R	agttcttctcccttacccatagtgtattacctttaataattaagtgtgcc
dnaKJ-F	gcggatcttccagagatttcttgtcctgccatatcgcg
dnaKJ-R	agttcttctcccttacccatctaaacgtctccactatatattcgg
csrA-F	gcgcggatcttccagagatttacctgcagcgttagccagtg
csrA-R	agttcttctcccttacccatagtgtattacctttaataattaagtgtgcc
*SDH*-R	gttcttctcccttacccattcaggcgttcccctgaatgaaatc
infC-rplT-*SDH*-F	aacaattggaggaataaggtatgacgagcggttttgattacatcg
lpp-*SDH*-F	caatctagagggtattaataatgacgagcggttttgattacatcg
cspA-*SDH*-F	attattaaaggtaatacactatgacgagcggttttgattacatcg
dnaKJ-*SDH*-F	tatatagtggagacgtttagatgacgagcggttttgattacatcg
csrA-*SDH*-F	aatctttcaaggagcaaagaatgacgagcggttttgattacatcg

### Culture Conditions

*E. coli* strains with plasmids are plated on LB plates with 100 mg/L of ampicillin and cultured overnight. A single colony is picked into a 14 mL shaker tube containing 4 ml LB medium and then cultured for 9–10 h as seed culture. The seed culture was then inoculated with an inoculation ratio of 2% into a 250 mL shake flask containing 25 mL of LB medium with 1% L-sorbose, firstly incubated at 37°C for 3 h, and then transferred to 25, 30, 37, and 40°C, respectively, for optimizing the temperature for enzyme expression.

### Simultaneous Overexpression of SDH and SNDH

For simultaneous overexpression of SDH and SNDH, the highest yielding constitutive promoter P_cspA_ was selected as pMD19-cspA-SDH. *E. coli* BL21(DE3) competent cell was simultaneously transfected into pMD19-cspA-SDH and pET28a-SNDH. Both SDH and SNDH were from *G. oxydans* WSH-004. Single colonies were picked into seed culture medium for 9 h, then transferred to a 250 mL shake flask containing 25 mL of LB medium with 1% L-sorbose by 2% inoculum, then incubated at 37°C for 2–3 h until about OD_600_ = 0.6–1, adding IPTG with a final concentration of 0.3 mM, transferring to a 30°C shaker for subsequent fermentation, taking samples at different times and used HPLC to detect for 2-KLG yield.

For optimizing sorbosone dehydrogenase (SNDH) from different sources, five SNDH from other sources were synthesize the five SNDH genes. To construct plasmids for overexpression of SNDH in *E. coli* BL21 (DE3), SNDH genes were inserted into the *Hin*dIII/*Eco*RI site of pET28(a)+. These plasmids containing SNDH werer transformed into *E. coli* BL21 (DE3) strain containing SDH plasmid to achieve the co-expression of SDH and SNDH in *E. coli*.

### Knockout of Aldosterone Reductase Genes

The aldosterone reductase genes, *yiaK, ahr, dkgA, dkgB, yahK, yajO, ydjG, yeaE*, have been amplified from genomic DNA of *E. coli* BL21 (DE3) by using *yiaK*-UP-F/R, *yiaK*-Down-F/R, *ahr*-UP-F/R, *ahr*-Down-F/R, *dkgA*-UP-F/R, *dkgA*-Down-F/R, *dkgB*-UP-F/R, *dkgB*-Down-F/R, *yahK*-UP-F/R, *yahK*-Down-F/R, *yajO*-UP-F/R, *yajO*-Down-F/R, *ydjG*-UP-F/R, *ydjG*-Down-F/R, *yeaE*-UP-F/R, *yeaE*-Down-F/R, respectively, to obtain upstream and downstream homology arm fragments. These genes of homologous arm were obtained by overlapping PCR using PrimerSTAR DNA polymerase. All the primers are listed in Table 2. The pCas plasmid can generate sgRNA located on the pTarget plasmid under IPTG induction, and the pCas plasmid itself will be incubated at 42°C. A series of pTarget plasmids were constructed containing the target gene N20-sgRNA of aldosterone reductase genes by primer sequences listed in Table 2. Knockout of these genes by CRISPR/Cas9 are performed according to the previous report (Jiang et al., 2015).

**Table 2:** Primers used for knockout aldosterone reductases.

**Primer**	**Sequence 5^**′**^-3^**′**^**
*yahK*-UP-F	ccagggagtggggcaatctgaatatg
*yahK*-UP-R	attatgtggcgcagctactgtattccgccccacaatttcatgacccggca
*yahK*-Down-F	tgccgggtcatgaaattgtggggcggaatacagtagctgcgccacataat
*yahK*-Down-R	ccaggcactatcagaaatcgctcat
*yahK*-sgRNA-F	gtggctcctttgttgtgtgcgttttagagctagaaatagcaagtt
*yahK-*sgRNA-R	gcacacaacaaaggagccacactagtattatacctaggactgagc
*dkgA*-UP-F	aggaggaacgtatggctaatccaac
*dkgA*-UP-R	cttccggctctgcatgatgatgtccggtgatggatctggaagttgcacac
*dkgA*-Down-F	gtgtgcaacttccagatccatcaccggacatcatcatgcagagccggaag
*dkgA*-Down-R	gctgccatgattgctgacaatatc
*dkgA*-sgRNA-F	ccattagcgcaaggagggaagttttagagctagaaatagcaagtt
*dkgA*-sgRNA-R	ttccctccttgcgctaatggactagtattatacctaggactgagc
*dkgB*-UP-F	gccagaatcgcaaaaatcctctgca
*dkgB*-UP-R	agaggcttaatcccattcaggagcccggccagtggattagagtcagatca
*dkgB*-Down-F	tgatctgactctaatccactggccgggctcctgaatgggattaagcctct
*dkgB*-Down-R	gcgctggtacgttaacggattcca
*dkgB*-sgRNA-F	gaagggttgacgcgtgagatgttttagagctagaaatagcaagtt
*dkgB*-sgRNA-R	atctcacgcgtcaacccttcactagtattatacctaggactgagc
*ahr*-UP-F	ggctggaacgcttaaatgatgcttc
*ahr*-UP-R	gcggtaatcagatcaactgcgagcacttatgagctgcgtaagctgatgcg
*ahr*-Down-F	cgcatcagcttacgcagctcataagtgctcgcagttgatctgattaccgc
*ahr*-Down-R	cgttgtggattatacctgtcgcacg
*ahr*-sgRNA-F	caaaatagggctgccagtcggttttagagctagaaatagcaagtt
*ahr*-sgRNA-R	cgactggcagccctattttgactagtattatacctaggactgagc
*yeaE*-UP-F	cgcgtgtatctgaatccacaaga
*yeaE*-UP-R	ggcattgaactcggtttaaccctcacaatatcagcgcggcacaagtattg
*yeaE*-Down-F	caatacttgtgccgcgctgatattgtgagggttaaaccgagttcaatgcc
*yeaE*-Down-R	cgggtattggtgtgcagtggaac
*yeaE*-sgRNA-F	atctgctgttgctggcaccagttttagagctagaaatagcaagtt
*yeaE*-sgRNA-R	tggtgccagcaacagcagatactagtattatacctaggactgagc
*yajO*-UP-F	tcggcgtctatctttgtcatcagac
*yajO*-UP-R	gtaatcacgcatggacactgccggaattatcggtacatcgcgggaagaac
*yajO*-Down-F	gttcttcccgcgatgtaccgataattccggcagtgtccatgcgtgattac
*yajO*-Down-R	tctggaaatggccaccagccatg
*yajO*-sgRNA-F	gcccggtttactcaacaaccgttttagagctagaaatagcaagtt
*yajO*-sgRNA-R	ggttgttgagtaaaccgggcactagtattatacctaggactgagc
*ydjG*-UP-F	tcatcttccagcttctcaagatcgc
*ydjG*-UP-R	cctttaggcacaacggatattacgc ccgctatgtcgtgataatggcattg
*ydjG*-Down-F	caatgccattatcacgacatagcgg gcgtaatatccgttgtgcctaaagg
*ydjG*-Down-R	acaaaccgtaacggcagtctgtggg
*ydjG*-sgRNA-F	agctggcttctacctcttcggttttagagctagaaatagcaagtt
*ydjG*-sgRNA-R	cgaagaggtagaagccagctactagtattatacctaggactgagc
*yiaK*-UP-F	ccatgtagatcttgcccattgcg
*yiaK*-UP-R	gcgcgatatgtccaaaaatcatgacctcaaatgtcactttcatcccaggc
*yiaK*-Down-F	gcctgggatgaaagtgacatttgaggtcatgatttttggacatatcgcgc
*yiaK*-Down-R	ttcaagtcgcatgtgcagcaacct
*yiaK*-sgRNA-F	ggcgcaaaagagtgtcgcatgttttagagctagaaatagcaagtt
*yiaK*-sgRNA-R	atgcgacactcttttgcgccactagtattatacctaggactgagc

### Knockout of Related Genes About L-Sorbose-Specific-PTS

By using the CRISPR/Cas9 technology, continued to knock out six related genes (*ptsG, fruA, pfkA, ptsH, ptsI, glcA*) in L-sorbose-specific PTS was based on the original strain *E. coli* BL21 (DE3) and *E. coli* BL21 (DE3)-8. A series of pTarget plasmids were constructed containing the target gene N20-sgRNA of aldosterone reductase genes by primer sequences listed in Table 3. Knockout of these genes by CRISPR/Cas9 are performed according to the previous report (Jiang et al., 2015). Using the *E. coli* BL21 (DE3) genome as a template, respectively, amplify using PrimerSTAR DNA polymerase, using *ptsG*-UP-F/R, *ptsG*-Down-F/R, *fruA*-UP-F/R, *fruA*-Down-F/R, *glcA*-UP-F/R, *glcA*-Down-F/R, *ptsI-ptsH*-UP-F/R, *ptsI-ptsH*-Down-F/R, as a pair primer to obtain upstream and downstream homology arm fragments. These genes of homologous arm were obtained by overlapping PCR using PrimerSTAR DNA polymerase. All the primers are listed in Table 3.

**Table 3:** Primers used for knockout of PTS related genes.

**Primer**	**Sequence 5^**′**^-3^**′**^**
*ptsG*-UP-F	actcaccttaccttgcgccggtacc
*ptsG*-UP-R	gtaccgaaaatcgcctgaacaccagaaccgcctgcttctgccataacatg
*ptsG*-Down-F	catgttatggcagaagcaggcggttctggtgttcaggcgattttcggtac
*ptsG*-Down-R	ggcgcaattaccgacaactggcagc
*ptsG*-sgRNA-F	tccttcatttggccgccgatgttttagagctagaaatagcaagtt
*ptsG-*sgRNA-R	atcggcggccaaatgaaggaactagtattatacctaggactgagc
*fruA*-UP-F	caacgccaggttttgtgcaatatt
*fruA*-UP-R	cggtaaaaatgtctggctgggtgatagcgaaagcagcgtaataaaaggtg
*fruA*-Down-F	caccttttattacgctgctttcgctatcacccagccagacatttttaccg
*fruA*-Down-R	tgaaacctaaccgccgcgagctgga
*fruA*-sgRNA-F	aaactgatggcaccacacgggttttagagctagaaatagcaagtt
*fruA-*sgRNA-R	ccgtgtggtgccatcagtttactagtattatacctaggactgagc
*glcA*-UP-F	cggcatcactgatagaaaaacaggtg
*glcA*-UP-R	gacatgtcgctggagcaataaccctatcgaccacggcgcagcaaatcaac
*glcA*-Down-F	gttgatttgctgcgccgtggtcgatagggttattgctccagcgacatgtc
*glcA*-Down-R	gcgaaagtggttgatcagcaaaacg
*glcA*-sgRNA-F	agaacggcacaagaaccgacgttttagagctagaaatagcaagtt
*glcA-*sgRNA-R	gtcggttcttgtgccgttctactagtattatacctaggactgagc
*pfkA*-UP-F	ttgatatcgtgacttcctggccggg
*pfkA*-UP-R	gctgttcgttctggatacctacgcacgctgtaacggtctagctgtaccat
*pfkA*-Down-F	atggtacagctagaccgttacagcgtgcgtaggtatccagaacgaacagc
*pfkA*-Down-R	actgttcgtacaattcgcgcgttgg
*pfkA*-sgRNA-F	gaagtgatgggccgttattggttttagagctagaaatagcaagtt
*pfkA-*sgRNA-R	caataacggcccatcacttcactagtattatacctaggactgagc
*ptsI-ptsH*-UP-F	gcaggtatctcttctggagcagctg
*ptsI-ptsH*-UP-R	ccagcgtcattaactcgtccgttgtagcggtaatggtaacttcttgctgg
*ptsI-ptsH*-Down-F	ccagcaagaagttaccattaccgctacaacggacgagttaatgacgctgg
*ptsI-ptsH*-Down-R	gcgaaagtggttgatcagcaaaacg
*ptsI-ptsH*-sgRNA-F	gttgtgactatctccgcagagttttagagctagaaatagcaagtt
*ptsI-ptsH-*sgRNA-R	tctgcggagatagtcacaacactagtattatacctaggactgagc

### Error-Prone PCR

Error-prone PCR of SDH was performed by the GeneMorph II Random Mutagenesis Kit (Agilent, Santa Clara, CA) with pMD19-cspA-SDH as a template by using primer pair 5′-TTCCAGAGATTATTGCTGTTTACGG-3′/5′-GTGAAAAGTTCTTCTCCCTTACCCA-3′. The amplified fragment carrying A tail was directly cloned with the vector pMD19-Simple transformed into *E. coli* BL21 (DE3) derivative strains mentioned above. For primary screening, the colonies appeared on the LB plates with ampicillin were picked up into 96-deep well plates by QPix420 (MD, Genetix, UK). These cultured cells in the deep-well plate were cultured at 37°C for 2–3 h, then moved to culture at 30°C for 20–24 h. For high-throughput screening, 40 uL of supernatant was transferred into another 96-well plate, then mixed with buffer containing 2-KLG reductase and NADH to a total volume 200 uL. The last one of each 96-well plate was set as a control. The absorbance at 340 nm, which is the optimum absorbance for NADH, was detected by a microplate reader (BioTek, Winooski, VT).

### HPLC and Liquid Chromatography Ion Trap Time-Of-Flight Mass Spectrometry (LCMS-IT-TOF) Assays

L-sorbose and 2-KLG were determined by a HPLC equipped with an Aminex HPX-87H column (Bio-Rad, Hercules, CA) at 35°C with a flow rate of 0.5 mL/min and 5 mmol/L H_2_SO_4_ as the eluent (Gao et al., 2014). For LC-MS analysis, a Shimadzu LCMS-IT-TOF (Shimadzu, Kyoto, Japan) equipped with an Aminex HPX-87H column was used. The HPLC conditions for LCMS-IT-TOF analysis is: 35°C with a flow rate of 0.5 mL/min and 5 mmol/L formic acid as the eluent. IT-TOF detection was performed with an ESI source in negative ion mode at the followed conditions: detector voltage, 1.60 kV; nebulizing gas (N2) flow, 1.5 L/min; drying gas (N2) flow, 200 kPa; ion accumulation time, 30 ms; and scan range (*m/z*), 100–300 for MS1(Chen et al., 2019).

## Results

### Overexpression of the FAD-Dependent L-Sorbose Dehydrogenase in *E. coli*

**Figure 1:**
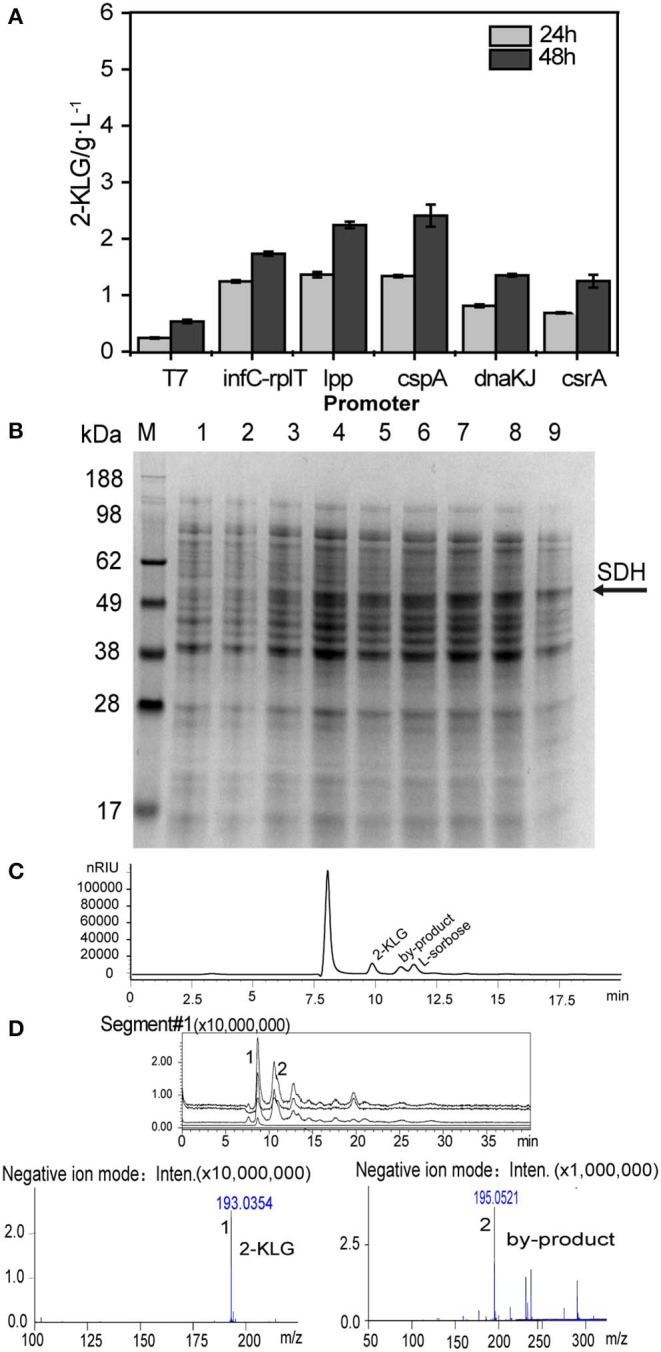
Optimized different constitutive promoter for the expression of SDH. **(A)** Effect of different promoter on 2-KLG production by HPLC analysis. **(B)** Whole cell SDS-PAGE analysis M: protein Marker, 1: blank control, and 2–9 represent constitutive expression of SDH 4, 6, 8, 10, 12, 16, 20, and 24h, respectively. **(C)** HPLC detection of product peaks **(D)** LC-MS analysis of product peaks.

Five *E. coli* BL21 (DE3) strains that express the FAD-dependent SDH with different promoters were constructed and applied for the shake flask culture to produce 2-KLG with 10 g/L of L-sorbose as a substrate. The sampled culture broth was then detected by HPLC (Figure 1A). T7 and five constitutive promoters were applied to optimize the expression of SDH in *E. coli*. The results showed that the promoter P_cspA_ could achieve to 2.42 g/L, which is the highest 2-KLG titer among the strains. SDS-PAGE results showed that the promoter P_cspA_ could significantly express SDH, which has a molecular weight of SDH was 57.6 kDa (Figure 1B). Since both the titer and the conversion ratio is relatively low, it cannot be directly applied for the high-throughput screening of the SDH. The HPLC and LC-MS results showed that besides the L-sorbose and 2-KLG, there is also an unknown byproduct (Figures 1C,D). This by-product was produced in the middle of the fermentation stage. As the fermentation progresses, the by-product gradually decreased. At the end of the fermentation, the by-product peak was almost disappeared.

### Optimized Production of 2-KLG From L-Sorbose

**Figure 2:**
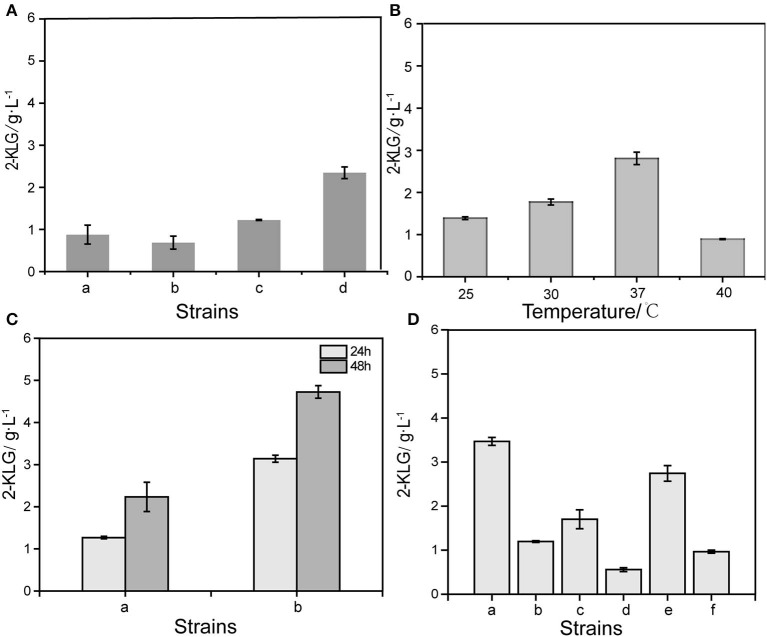
Optimized different conditions of 2-KLG yield from L-sorbose. **(A)** Optimized host cells for expression of sorbose dehydrogenase. a: *E.coli* JM109, b: *E.coli* K-12substr.W3110, c: *E.coli* K-12substr.MG1655, d: *E.coli* BL21(DE3). **(B)** Optimized the effect of culture temperature on the yield of 2-KLG. **(C)** Effect of co-expression of SDH/SNDH on 2-KLG production. a: Control, b: T-cspA-SDH/pET28a-SNDH. **(D)** Optimized the effects of different sources of sorbosone dehydrogenase (SNDH) on the yield of 2-KLG. a: sndh-WSH-004, b: sndh-02655, c: sndh-02935, d: sndh-03750, e: sndh-04500, f: sndh-19405.

By expressing the SDH with the optimum promoter P_cspA_ in different *E. coli* strains, it was found that SDH could convert L-sorbose to 2-KLG in *E. coli* K-12 substr. W3110, *E. coli* K-12 substr. MG1655, *E. coli* JM109 and *E. coli* BL21 (DE3) (Figure 2A). The result showed that the SDH could yield highest 2-KLG titer in *E. coli* BL21 (DE3), while the 2-KLG titers in the other three strains are low. Then the *E. coli* BL21 (DE3) was selected as the host for the downstream experiments. The *E. coli* BL21 (DE3) strain with the plasmid pMD19-cspA-SDH was subjected to shake flask fermentation at 4 different temperatures to obtain the optimum temperature for enzyme expression (Figure 2B). The results showed that the optimum temperature for 2-KLG production was 37°C, under which the 2-KLG titer could reached to 2.83 g/L. Furthermore, when simultaneous expression of pMD19-cspA-SDH and pET28a-SNDH in *E. coli* BL21 (DE3), the 2-KLG titer could be further improved to 2.54 g/L at 48 h (Figure 2C). Base on the results, different SNDH have been co-expressed. The results showed that co-expression of SNDH from WSH-004 could achieve the highest 2-KLG titer (Figure 2D).

### Effects of Aldosterone Reductases Knockout on 2-KLG Production in *E. coli*

By knocking out the 8 aldosterone reductase gene using the CRISPR/Cas9 technology, an *E. coli* strain without aldosterone reductases, *E. coli* BL21(DE3)-8 (Table 4) has been obtained. Then the SDH or SDH/SNDH was transferred into the aldosterone reductases defective strain to form *E. coli* BL21(DE3)-8-SDH and *E. coli* BL21(DE3)-8-SDH-SNDH. By culture of *E. coli* BL21(DE3)-8-SDH in shake flasks using 10 g/L of L-sorbose as the substrate, the 2-KLG titer was slightly decreased from 2.58 to 2.32 g/L, while the 2-KLG titer of *E. coli* BL21(DE3)-8-SDH-SNDH was increased from 4.43 to 5.85 g/L. The results suggested that the aldosterone reductases could play important roles in the function of SDH and SNDH.

**Table 4:** Strains deficient in aldosterone reductases and PTS system.

**Strains**	**Genotypes**
*E. coli* BL21 (DE3)-3	Δ *ptsG:* Δ *fruA:* Δ *glcA*
*E. coli* BL21 (DE3)-4	Δ *ptsG:* Δ*fruA:* Δ *glcA:* Δ *pfkA*
*E. coli* BL21 (DE3)-6	Δ *ptsG:* Δ*fruA:* Δ *glcA:* Δ *pfkA* Δ*ptsH:* Δ*ptsl*
*E. coli* BL21 (DE3)-8	Δ*dkgB:* Δ *ahr:* Δ *yajO:* Δ *yiaK:* Δ *yahK:* Δ *ydjG:* Δ *yeaE:* Δ*dkgA*
*E. coli* BL21(DE3)-8-SDH	Δ*dkgB:* Δ *ahr:* Δ *yajO:* Δ *yiaK:* Δ *yahK:* Δ *ydjG:* Δ *yeaE:* Δ*dkgA*, SDH
*E. coli* BL21(DE3)-8-SDH-SNDH	Δ*dkgB:* Δ *ahr:* Δ *yajO:* Δ *yiaK:* Δ *yahK:* Δ *ydjG:* Δ *yeaE:* Δ*dkgA*, SDH, SNDH
*E. coli* BL21 (DE3)-11	Δ*dkgB:* Δ*ahr*: Δ*yajO*: Δ*yiaK*: Δ*yahK*: Δ*ydjG*: Δ*yeaE*: Δ*dkgA*: Δ*ptsG*: Δ*fruA*: Δ*glcA*
*E. coli* BL21 (DE3)-12	Δ*dkgB:* Δ*ahr*: Δ*yajO*: Δ*yiaK*: Δ*yahK*: Δ*ydjG*: Δ*yeaE*: Δ*dkgA*: Δ*ptsG*: Δ*fruA*: Δ*glcA*: Δ *pfkA*
*E. coli* BL21 (DE3)-13	Δ*dkgB:* Δ*ahr*: Δ*yajO*: Δ*yiaK*: Δ*yahK*: Δ*ydjG*: Δ*yeaE*: Δ*dkgA*: Δ*ptsG*: Δ*fruA*: Δ*glcA*: Δ *ptsH*: Δ *ptsl*
*E. coli* BL21 (DE3)-14	Δ*dkgB:* Δ*ahr*: Δ*yajO*: Δ*yiaK*: Δ*yahK*: Δ*ydjG*: Δ*yeaE*: Δ*dkgA*: Δ*ptsG*: Δ*fruA*: Δ*glcA*: Δ *pfkA:* Δ *ptsH*: Δ *ptsl*

### Effects of PTS System Proteins Knockout on 2-KLG Production in *E. coli*

**Figure 3:**
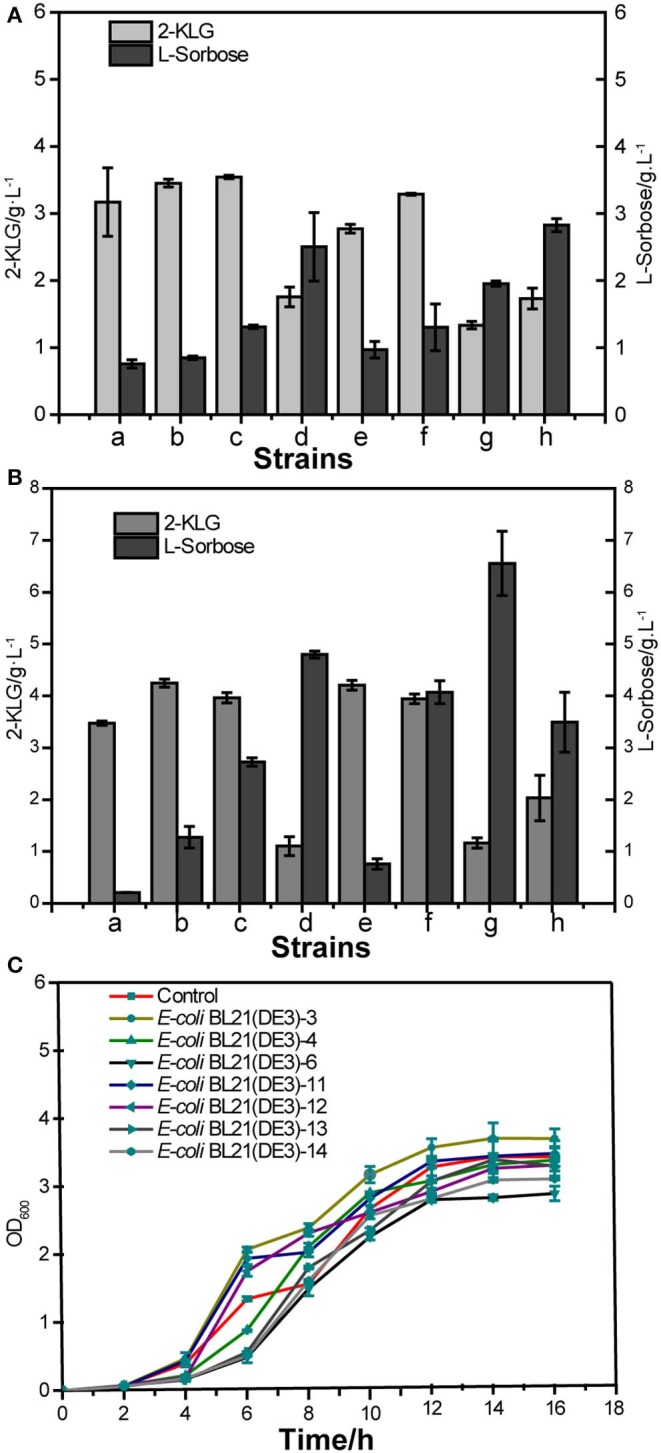
Effects of PTS system proteins knockout on 2-KLG production in *E. coli*. **(A)** Overexpression of sorbose dehydrogenase (SDH) in defective strains. a: WT *E-coli* BL21(DE3), b: *E-coli* BL21(DE3)-3, c: *E-coli* BL21(DE3)-4, d: *E-coli* BL21(DE3)-6; e: *E-coli* BL21(DE3)-11, f: *E-coli* BL21(DE3)-12, g: *E-coli* BL21(DE3)-13, h: *E-coli* BL21(DE3)-14. **(B)** Co-expressed sorbose dehydrogenase and sorbosone dehydrogenase (SDH/SNDH) in defective strains. a: Control, b: *E-coli* BL21(DE3)-3, c: *E-coli* BL21(DE3)-4, d: *E-coli* BL21(DE3)-6; e: *E-coli* BL21(DE3)-11, f: *E-coli* BL21(DE3)-12, g: *E-coli* BL21(DE3)-13, h: *E-coli* BL21(DE3)-14. **(C)** Defective strain cell growth OD_600_ detection.

Seven PTS system proteins knockout strains were constructed to evaluate the effect of these proteins on the 2-KLG production, i. e., *E. coli* BL21 (DE3)-3, *E. coli* BL21 (DE3)-4, *E. coli* BL21 (DE3)-6, *E. coli* BL21 (DE3)-11, *E. coli* BL21 (DE3)-12, *E. coli* BL21 (DE3)-13, *E. coli* BL21 (DE3)-14 (Table 4). Then SDH or SDH/SNDH were overexpressed in these strains, respectively. The results of shake flask fermentation showed that knockout of *ptsH* and *ptsI* could significantly improve the 2-KLG titer in strains either overexpression of SDH or SDH/SNDH (Figures 3A,B). The results also showed that knocking out *ptsH* and *ptsI* had a great effect on the growth of the cells in the early stage, while the cell growth could achieve the similar level to that of the control strain (Figure 3C).

### High Throughput Screening of Sorbose Dehydrogenase in *E. coli*

**Figure 4:**
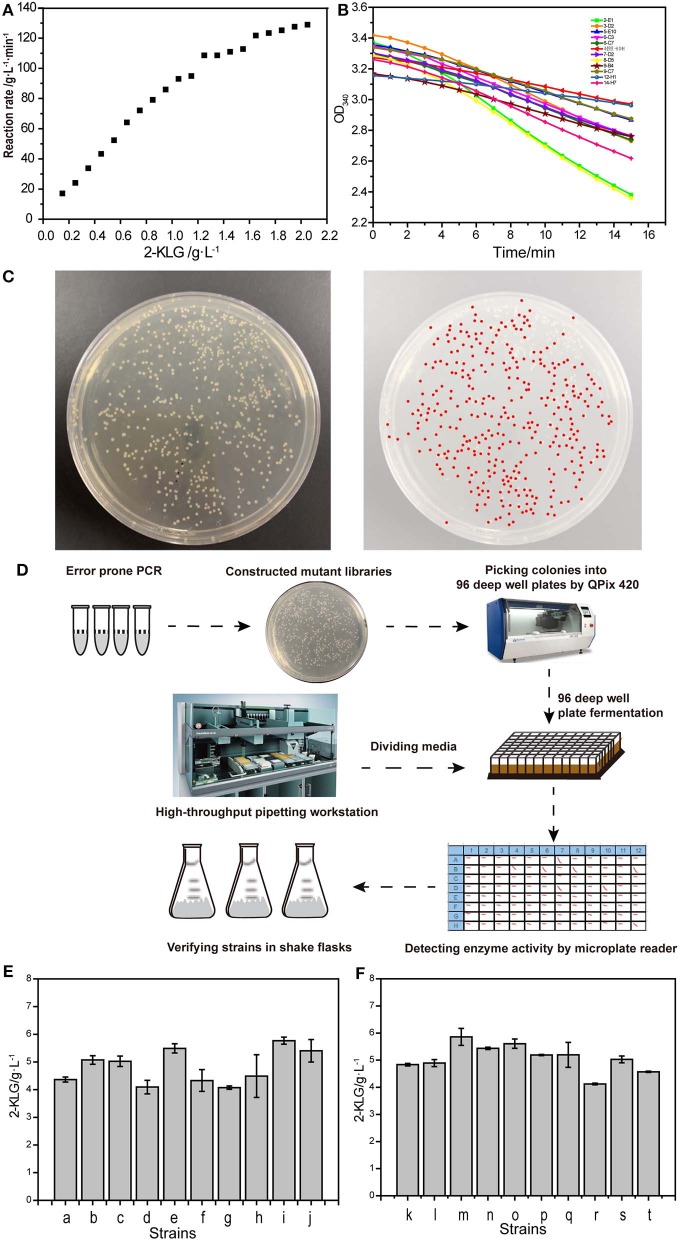
High-throughput screening system. **(A)** Reaction rate of 2-KLG reductase with different concentrations of 2-KLG. **(B)** High-throughput preliminary screening visual map based on microplate reader. **(C)** A typical colony EP-PCR result. **(D)** The workflow of high-throughput screening method. **(E)** Verify of the strains in shake flasks (batch 1). a: Control, b: co-2-E1, c: co-8-D5, d: co-9-B4, e: co-6-F7, f: co-6-C3, g: co-5-E10, h: co-3-D2, i: co-15-A7, j: co-16-B6. **(F)** Verify of the strains in shake flasks (batch 2). k: Control, l: co-20-A1, m: co-28-B1, n: co-19-C1, o: co-15-D1, p: co-33-E1, q: co-6-F1, r: co-8-G1, s: co-25-H1, t: co-5-H5.

It was determined that the optimal limited range for detection of 2-KLG with 2-KLG reductase was 0–0.23 (g/L) (Figure 4A). A series of mutants was obtained by using the mutation kit. The initial screening was performed by using microplate reader (Figure 4B). Around 1.3 × 10^4^ mutants were screened for each round. Figure 4C showed one typical colony EP-PCR result by using the workflow shown in Figure 4D. The optimum mutants obtained by preliminary screening were subjected for rescreening with shake flasks. Six strains with higher 2-KLG titer was obtained by rescreening (Figures 4E,F), namely co-2-E1, co-16-B6, co-8-D5, co-6-F7, co-15-A7, co-28-B1, increased by 7.1, 10.4, 6.6, 11.3, 14.1, and 10.2%, respectively. The specific amino acid mutations of these beneficial mutants were shown in Table 5. The results showed that the method established here can be used for high-throughput screening of enhanced SDH. In theory, when the GeneMorph II Random Mutagenesis Kit could yield the similar the probability of mutation of the target gene to A, T, G, and C base. However, 80% of the target gene was mutated to A or T base in the screened SDH, indicating that the gene may have a preference for A or T bases.

**Table 5:** Mutation sites in SDHs.

**Mutants**	**Mutation sites**
co-2-E1	Cys17Arg, Gly134Asp, Ile234Val, Val280Met, Trp316Arg, Thr325Asn
co-16-B6	Lys313Glu, Glu274Ile
co-8-D5	Leu44Gln, Glu66Gly, Tyr78Phe, Gly141Val, Asn147Ile, Val159Ala, Ile221Phe, Arg273His, Thr304Ala, Asp407Val, Val490Asp
co-6-F7	Thr443Ile, Gly276Asp
co-15-A7	His59Gln, Glu243Gly, Met326Leu, Ser349Pro
co-28-B1	Lys53Arg, Thr65Ala, Thr96Ala, Asp118His, Val119Asp, Ser146Thr, Asn152Thr, Ile234Asn, Asn236Asp, Ser348Thr, Gly377Trp, Ser510Thr

## Discussion

SDH with high substrate/product specificity and high enzyme activity is vital for achieving one-step-single-strain production of 2-KLG from D-sorbitol. A FAD-dependent SDH obtained by our previous work has high substrate/product specificity while low enzyme activity was used to improve the enzyme activity by high-throughput screening. By optimizing the promoter, hosts and SNDHs, knockout of the aldosterone reductases and PTS related genes, a reliable platform for high-throughput screening of more efficient FAD-dependent SDH has been established. By using the high-throughput screening system, the titer of the 2-KLG has been improved by 14.1%.

At present, most of the recent researches on the one-step-single-strain production of L-Asc were carried out in the *G. oxydans* (Table 6) (Wang et al., 2018). The SDH has a similar sequence to that of the *G. oxydans* T100, which could directly convert D-sorbitol to 2-KLG (Saito et al., 1997). When overexpression of the SDH from *G. oxydans* T100 in another *G. oxydans* G624, the titer of 2-KLG could achieve to 130 g/L (Saito et al., 1997). However, when synthesize the SDH and SNDH from *G. oxydans* T100 in *G. oxydans* strains available in our lab, no more than 10 g/L of 2-KLG could be obtained. Unlike a majority of the common enzymes, these SDHs from *G. oxydans* are highly hypercritical to strains without any known disciplines according to our experiments, such as: (1) The SDHs from one *G. oxydans* strain cannot be functional in other *G. oxydans* strains and other common bacteria; (2) *E. coli* strains express the SDHs could have the ability to convert L-sorbose to 2-KLG, while the broken cells cannot, even with common exogenous electron acceptors for dehydrogenases (DCIP, PMS). These strange phenomena significantly affect the further rational engineering of the SDHs from *G. oxydans*. Since the slow growth rate of *G. oxydans* and low transformation efficiency (Yao et al., 2017; Jin et al., 2019), *G. oxydans* itself is not suitable for high-throughput screening of efficient SDH. *E. coli* could be a common host for the high-throughput screening of enzymes by using error-prone PCR.

**Table 6:** Production of -KLG by different microorganisms.

**Microorganisms (year)**	**Substrate**	**Concentration (g/L)**	**Titer (g/ L)**	**Yield** **(mol/ mol)**
*G. oxydans* T-100 (1997)	D-Sorbitol	50	7.0	13.1%
*P.putida* IFO3738 (2001)	D-Sorbitol	50	11.6	21.8%
*G.oxydans* (pGUC-k0203-GS-k0095-pqqABCDE) (2014)	D-Sorbitol	150	39.2	24.5%
*G. oxydans*-ss-pqqABCDE (2016)	D-Sorbitol	150	44.5	27.8%
*G. melanogenus* Z84 (1990)	D-Sorbitol	100	60.0	55.7%
*G. oxydans* NB6939 (pSDH-tufB1) (1997)	D-Sorbitol	150	130.0	81.3%

By optimizing the promoters and the host of *E. coli*, it was found that not only the conversion ratio of L-sorbose to 2-KLG was low, there was also some byproducts that may be associated with the SDH. In order to decrease the accumulation of by-product, several means have been attempted, such as coexpression of SNDH (Fu et al., 2007; Du et al., 2013), blocked the potential competition pathway of L-sorbose to 2-KLG by knocking out the aldosterone reductase genes (Yum et al., 1998; Penning, 2015). According to reports in the gene of *Klebsiella*, the transport of L-sorbose into bacteria requires the L-sorbose-specific phosphotransferase system (PTS) (Slater et al., 1981; Wehmeier et al., 1995; Yebra et al., 2000). Therefore, knockout of PTS related genes in *E. coli* has also been attempted, including PtsG (glucose phosphoryl transferase) (Han et al., 2004), FruA (fructose phosphoryl transferase), PfkA (phosphofructokinase) (Vinopal et al., 1975), PtsH (phosphorylated carrier protein), PtsI (phosphotransferase I) (Woodward and Charles, 1982), and GlcA (glycolate transporter) (Sprenger and Lengeler, 1984). Though the interactions of SDH with these genes remains unclear, our results demonstrated that knockout of some of the genes could significantly enhance the function of SDH in *E. coli*, which could benefit the high-throughput screening of enhanced SDH in *E. coli*.

After investigated a majority of the SDHs reported in *G. oxydans* in our lab, the enzyme activity in either *G. oxydans* or *E. coli* is far away from the requirement for competition with the current industrial scale vitamin C process. Though it has been reported that the *G. oxydans* strain can be directly mutagenized to enhance its performance in 2-KLG production (Park et al., 2012; Zhu et al., 2012; Yang et al., 2017), there was no reports on the directed evolution of SDH from *G. oxydans*. In this study, it was found that only overexpression of SDH in *E. coli* BL21 (DE3) could produce 2-KLG. Though the current mixed fermentation system for the production of 2-KLG has been extensively studied (Takagi et al., 2010; Yang et al., 2015), production of 2-KLG in *E. coli* are rarely investigated. The results presented here could provide a useful reference for the metabolic engineering of L-sorbose to 2-KLG in *E. coli*. In this work, overexpression of a FAD-dependent SDH from *G. oxydans* WSH-004 in *E. coli* was studied. Owing to its unique characteristics, this SDH could be used to directly produce 2-KLG from L-sorbose with high substrate/product specificity. A platform strain suitable for high-throughput screening of SDH was constructed. The screening platform strain constructed here should be a model strain suitable for further enhancing the production of 2-KLG by either *G. oxydans* or other bacteria.

## Data Availability Statement

The raw data supporting the conclusions of this article will be made available by the authors, without undue reservation, to any qualified researcher.

## Author Contributions

JZ and JC provided the main core ideas of the experimental design. WZ, LL, and XS mainly searched for references, designed of experimental methods, purchase of consumables and reagents used in the experimental process, operated experiment and analysis of data, drawing of charts, and writing of articles.

### Conflict of Interest

The authors declare that the research was conducted in the absence of any commercial or financial relationships that could be construed as a potential conflict of interest.
